# HDAC Inhibition Induces PD-L1 Expression in a Novel Anaplastic Thyroid Cancer Cell Line

**DOI:** 10.1007/s12253-020-00834-y

**Published:** 2020-06-26

**Authors:** Luca Hegedűs, Dominika Rittler, Tamás Garay, Paul Stockhammer, Ildikó Kovács, Balázs Döme, Sarah Theurer, Thomas Hager, Thomas Herold, Stavros Kalbourtzis, Agnes Bankfalvi, Kurt W. Schmid, Dagmar Führer, Clemens Aigner, Balázs Hegedűs

**Affiliations:** 1 Department of Thoracic Surgery, University Medicine Essen - Ruhrlandklinik, University Duisburg-Essen, Essen, Germany; 2 2nd Department of Pathology, Semmelweis University, Budapest, Hungary; 3 Faculty of Information Technology and Bionics, Pázmány Péter Catholic University, Budapest, Hungary; 4 Department of Thoracic Surgery, Medical University of Vienna, Vienna, Austria; 5 National Korányi Institute of Pulmonology, Budapest, Hungary; 6 Department of Thoracic Surgery, Semmelweis University–National Institute of Oncology, Budapest, Hungary; 7 Institute of Pathology, University Clinic Essen, University Duisburg-Essen, Essen, Germany; 8 Department of Endocrinology, University Clinic Essen, University Duisburg-Essen, Essen, Germany

**Keywords:** Anaplastic thyroid cancer, BRAF mutation, TERT promoter mutation, HDAC inhibition, Pleural effusion

## Abstract

While papillary thyroid cancer (PTC) has largely favorable prognosis, anaplastic thyroid cancer (ATC) is a rare but extremely aggressive malignancy with grim clinical outcome. Even though new therapeutic options are emerging for ATC, additional preclinical models and novel combinations are needed for specific subsets of patients. We established a novel cell line (PF49) from the malignant pleural effusion of a 68-year-old male patient with ATC that rapidly transformed from a BRAF and TERT promoter mutant PTC. PF49 cells demonstrated a robust migratory activity in vitro and strong invasive capacity in vivo in a pleural carcinosis model. Combined BRAF and MEK inhibition decreased the proliferation and migration of PF49 cells, however could not induce cell death. Importantly, HDAC inhibitor treatment with SAHA or valproic acid induced cell cycle arrest and strongly increased PD-L1 expression of the tumor cells. Induction of PD-L1 expression was also present when paclitaxel-cisplatin chemotherapeutic treatment was combined with HDAC inhibitor treatment. Increased PD-L1 expression after HDAC inhibition was recapitulated in an international ATC cell model. Our data suggest that HDAC inhibition alone or in combination with standard chemotherapy may potentiate anaplastic thyroid cancer cells for immunotherapy.

**Electronic supplementary material:**

The online version of this article (10.1007/s12253-020-00834-y) contains supplementary material, which is available to authorized users.

## Background

Anaplastic thyroid cancer (ATC) is a rare but highly lethal disease that represents 1–2% percent of thyroid cancer cases but it is responsible for around 30% of thyroid cancer related death and the 5-year relative survival rate is around 10% [1, 2]. It mostly develops from follicular derived differentiated thyroid cancers or directly from normal follicular thyroid cells [3]. In rare occasions, a papillary thyroid carcinoma can also transform to an anaplastic carcinoma as it was demonstrated in several case studies [4, 5]. ATC has a heterogeneous mutational background. Mutation in the BRAF gene was described in 25% of the cases, while the prevalence of RAS gene mutations is around 10–25% [6]. TERT promoter mutation is also present in around 40% of the tumors [7, 8] and shows association with both the presence of BRAF mutation and advanced patient age [9]. Mutations in several tumor suppressor genes were also described and among these TP53 is the most common (25–60%), while mutations in PTEN and NF2 were found in lower frequencies [3]. This diverse genetic background promotes tumor aggressiveness, invasiveness and results in resistance to therapy.

Treatment of ATC is primarily surgery, when it is possible, together with pre- and postoperative radiotherapy and chemotherapy. Currently, there is no established standard-of-care chemotherapy for ATC, most frequently doxorubicin, paclitaxel and cisplatin are used [10]. However, none of these therapies could significantly improve clinical outcome [11]. For patients with metastatic or unresectable ATC with BRAFV600E mutation, combination treatment with mutant BRAF inhibitor dabrafenib and MEK inhibitor trametinib was recently approved by the FDA [12]. Furthermore, several other targeted and immunotherapeutic agents were or are currently tested in clinical trials. Multitarget tyrosine kinase inhibitors, such as sorafenib or lenvatinib, had a beneficial effect on patient overall survival and are already approved drugs for other thyroid tumor types [11]. Since ATCs frequently express PD-L1, the use of PD-L1/PD-1 inhibitors was also suggested as potential therapeutic option in ATC [13–15]. Combination of checkpoint inhibitor immunotherapy with BRAF inhibitor was found to be effective in a BRAF V600E mutant mouse ATC model [16]. Furthermore, in a patient with BRAF mutant ATC, BRAF and MEK inhibitors were successfully used together with PD-1 blocker pembrolizumab in a neoadjuvant setting enabling a complete surgical resection [17]. Pembrolizumab was also suggested as a salvage therapy for ATC patients who show progression after kinase inhibitor therapy [18].

Histone acetylation is often modified in cancer cells and certain histone deacetylase (HDAC) inhibitors are already used in other cancer types. Suberoylanilide hyroxamic acid (SAHA) is a broad range HDAC inhibitor and is FDA approved for cutaneous T cell lymphoma [19]. Its anti-tumor effect was described in several cancer types and it is currently tested in numerous clinical trials both as a single agent or in combination with other anti-neoplastic drugs [20, 21]. It induces cell cycle arrest and apoptosis in tumor cells and can inhibit angiogenesis and immunosuppressive interleukin production [22, 23]. Valproic acid is another HDAC inhibitor that is extensively tested in cancer treatment [24]. It is a small branched fatty acid that has been safely used as a drug to treat epileptic seizures and as a mood stabilizer for decades [25]. While as a single agent, a high dose of valproic acid is required to reach higher anti-cancer efficacy, it is tested in combination with cytotoxic agents [26]. In poorly differentiated thyroid cancer cells valproic acid treatment was shown to induce cell cycle arrest and apoptosis and it increased the sensitivity of the tumor cells to both doxorubicin and paclitaxel treatment [24, 27, 28]. In a case study, successful treatment of a patient with ATC was described by combined therapy of valproic acid, cisplatin and doxorubicin together with radiation and surgery [28]. However, when the combined effect of valproic acid and paclitaxel was compared with paclitaxel alone in a phase II/III clinical trial no advantage was found [29]. Pan HDAC inhibitor panobinostat is an FDA approved drug in third line against multiple myeloma and it also has a strong cytotoxic effect in ATC and squamous thyroid carcinoma cells through induction cell cycle arrest and apoptosis [30, 31].

ATC is highly invasive and it metastasizes most frequently to the lung and to the intrathoracic and neck lymph nodes [32]. In in vitro and in vivo studies a number of agents were identified that decreased migratory and metastatic capacity of ATC cells. In TERT promoter mutated cells silencing of hTERT strongly reduced the proliferation and migration of the cells [33]. Treatment with HDAC and EGFR dual inhibitor CUDC-101reduced tumor growth and metastases and increased survival in a metastatic ATC mouse model [34]. EGFR and VEGFR dual inhibitor AEE788 decreased angiogenesis in xenograft ATC tumors and this effect was further increased when AEE788 was used in combination with paclitaxel [35].

In the current study we describe a new anaplastic thyroid cancer cell line (PF49) derived from the malignant pleural effusion of a 70-year-old male patient. At the time of diagnosis the patient had a papillary thyroid cancer that already carried a BRAF(V600) and a TERT promoter mutation. We found that PF49 cells have a strong migratory capacity in vitro and they are highly invasive in vivo. Treatment with mutant BRAF inhibitor or MEK kinase inhibitor decreased the viability, proliferation and migration of the tumor cells and this effect was further increased by the combination of the two agents, but it did not induce cell death. HDAC inhibitor treatment caused cell cycle arrest but it did not affect cell migration. Importantly, HDAC inhibition increased the PD-L1 expression of the tumor cells both alone and in combination with conventional chemotherapy.

## Materials and Methods

### Cell Culture and Reagents

A375 is a melanoma cell line with a BRAF V600E mutation and it was obtained from ATCC. BHT-101 is an ATC cell line with a BRAF V600E and a TERT promoter mutation, it was purchased from DSMZ. Both were maintained in DMEM supplemented with 10% fetal bovine serum and 1% penicillin-streptomycin in tissue culture flasks at 37 °C and 5% CO_2_ in a humidified atmosphere.

PF49 cell line was established from a malignant pleural effusion. Briefly, 5 ml of effusion was centrifuged at 1000×g for 10 min. Following the removal of supernatant, the pellet was resuspended in RPMI1640 containing 10% FBS, and 100 U/ml penicillin-streptomycin. The suspension was plated in a tissue culture flask. The adherent cells were cultured for 6 passages in order to obtain a tumor cell culture free of non-tumor cells before experiments were performed. The study was approved by the Ethic Committee of the University Duisburg-Essen (#18–8208-BO) and the patient provided informed consent. The whole study was carried out in accordance with the Declaration of Helsinki. A375 and PF49 cells were subjected to Multiplex Cell Line Authentication (Multiplexion, Heidelberg, Germany).

Vemurafenib, selumetinib and dabrafenib were purchased from Selleck Chemicals, and were dissolved in DMSO and kept at −80 °C. From valproic acid sodium salt (Sigma-Aldrich) a stock solution was prepared in distilled water at 200 mM concentration, while SAHA (Sigma-Aldrich) was dissolved in DMSO at 100 mM concentration. Both were stored at −20 °C. Cisplatin (Accord, 1 mg/ml) and Paclitaxel (Kabi, 6 mg/ml) were stored at room temperature.

### Proliferation Assay

A375 and PF49 cells were seeded in a 50,000 cells / well concentration on 6-well plates. After 2, 4 and 6 days cells were counted with the NucleoCounter NC-3000™ system (Chemometec). For each time point cells were seeded in triplicates.

### Viability Assay

PF49 cells were seeded in 6-well plates and treated with paclitaxel (10 nM) or cisplatin (3 μM) alone and in combination with valproic acid (1 mM) or SAHA (1 μM) for 72 h. Then cells were trypsinized and viability was measured with NucleoCounter NC-3000™ system (Chemometec). Briefly, cells were stained with acridine orange and DAPI concomitantly; DAPI could only penetrate the dead cells.

### Time-Lapse Video Microscopy

Video microscopy measurements were performed and analyzed as described previously [36]. Briefly, cells were seeded in 24-well plates (Corning Incorporated, USA) and incubated overnight in DMEM medium supplemented with 10% FCS. CO_2_-independent culture medium (Gibco-BRL Life Technologies, UK) supplemented with 10% FCS and 4 mM glutamine was applied and the plate was transferred to the custom designed incubator built around an inverted phase-contrast microscope (World Precision Instruments, USA). The experiment was performed at 37 °C in room ambient atmosphere. Images were taken every 10 mins from three neighboring microscopic fields in each well for 72 h. Migration data was captured with a manual cell-tracking program. The parameter migrated distance is calculated by averaging for each cell the displacement for the 48–72 h period after treatment, in at least three microscopic fields.

### Sulforhodamine B (SRB) Assay

Cell viability was analyzed based on total protein amount with Sulforodamine B (SRB) assay. We seeded 3500 cells on the inner 60 wells of a 96 well-plate. After 24 h fresh medium was applied with various drug concentrations. 72 h later the medium was removed and the cells were fixed with 10% TCA. After that SRB dye was added and plates were incubated for 15 min. After several washes with 1% acetic acid to remove excess dye, we dissolved the protein-bound dye with 10 mM Tris puffer and measured the OD at 570 nm with a microplate reader (EL800, BioTec Instruments, Winooski, VT). Measurements were repeated three times and each time in triplicates, results are expressed as relative to control. Interactions between drugs were analyzed by calculating the combination index (CI) as in Chou and Talalay [37] with CompuSyn software (ComboSyn Inc). CI values CI > 1, CI = 1 or CI < 1 represents antagonism, additive effects, and synergism, respectively.

### Cell Cycle Analysis

The ratio of cells in each cell cycle phases was analyzed based on the DNA content of the cells. PF49 cells were seeded on 6-well plates in 1.5 × 10^5^ cells / well concentration. Treatments were performed for 72 h. First cells were trypsinized and then incubated with lysis buffer containing DAPI for 5 min at 37 °C. After that stabilization buffer was added to the samples and cellular fluorescence was measured by the NucleoCounter NC-3000system (Chemometec).

### Western Blot Analysis

Cells were precipitated with 6% ice-cold TCA for an hour and then centrifuged 10 min at 9000 rpm. Total cellular protein pellets were resuspended in electrophoresis sample buffer (62.5 mM Tris–HCl, pH 6.8, 2% SDS, 10% glycerol, 5 mM EDTA, 125 mg/mL urea, 100 mM dithiothreitol) and equal amounts of protein were loaded on 10% acrylamide gels. The following primary antibodies were used: rabbit monoclonal anti-phospho-p44/42MAPK (ERK1/2) (Cell Signaling, 4370S, dilution 1:2000), mouse monoclonal anti-ERK1/2 (Cell Signaling, 4696S, dilution 1:2000), rabbit polyclonal anti-beta-tubulin (Abcam, ab6046, 1:2000),, rabbit polyclonal anti-PD-L1 (Cell Signaling, 13,684 T, 1:1000). Then HRP-conjugated anti-rabbit and anti-mouse secondary antibodies (Jackson ImmunoResearch, dilution 1:10000) were applied and for detection Pierce ECL Western Blotting Substrate (Thermo Scientific) and luminography was used.

### In Vivo Tumorigenicity

The pleural carcinosis model was established by inoculating 200,000 cells in 100 μl DMEM into the thoracic cavity of six 12-week-old SCID mice. The animals’ weight was measured three times per week. When animals lost more than 15% of their weight, mice were sacrificed by cervical dislocation and the thoracic cavity was opened and photographed. Macroscopic tumor nodules, the lungs and the heart were collected, fixed in 4% paraformaldehyde and embedded in paraffin. The animal model protocol was carried out in accordance with the Guidelines for Animal Experiments and was approved for the National Institute of Oncology, Budapest, Hungary (PEI/001/2574–6/2015).

### Immunohistochemistry

Ventana BenchMark Ultra automated staining system (Roche Tissue Diagnostics, Grenzach-Vyhlen, Germany) was used for immunohistochemistry. From the formalin-fixed and paraffin embedded (FFPE) tumor specimen 3 μm sections were prepared. The following primary antibodies were used: anti-vimentin (Dako #M0725), anti-TTF1 (Dako, clone 8G7G3/1), anti-PAX8 (Cell Marque, MRQ-50), Cytokeratin 18 (Dako, Clone DC 10) and anti-thyroglobulin (Dako, A0251) and anti-PD-L1 (22C3, Dako). Antibody binding was detected with the UltraVision LP Detection System (Lab Vision Corporation). Color development with OptiView staining kit (Roche Tissue Diagnostics, Grenzach-Vyhlen, Germany) was followed by hematoxylin counterstaining. PD-L1 expression was quantified by a pathologist using the combined positive score (CPS).

### Statistics

Two-way ANOVA followed by Bonferroni posttest was used to establish whether significant differences existed between cell lines and treatment groups. One-way ANOVA followed by Tukey’s post hoc test or Dunn’s multiple comparison test were used to establish whether significant differences existed between treatment groups. Significant differences were indicated as *P < 0.05, **P < 0.01, ***P < 0.001. All statistical analyses were performed in GraphPad Prism 5 (GraphPad Software Inc., USA, San Diego, CA).

## Results

### Patient History

**Fig. 1:**
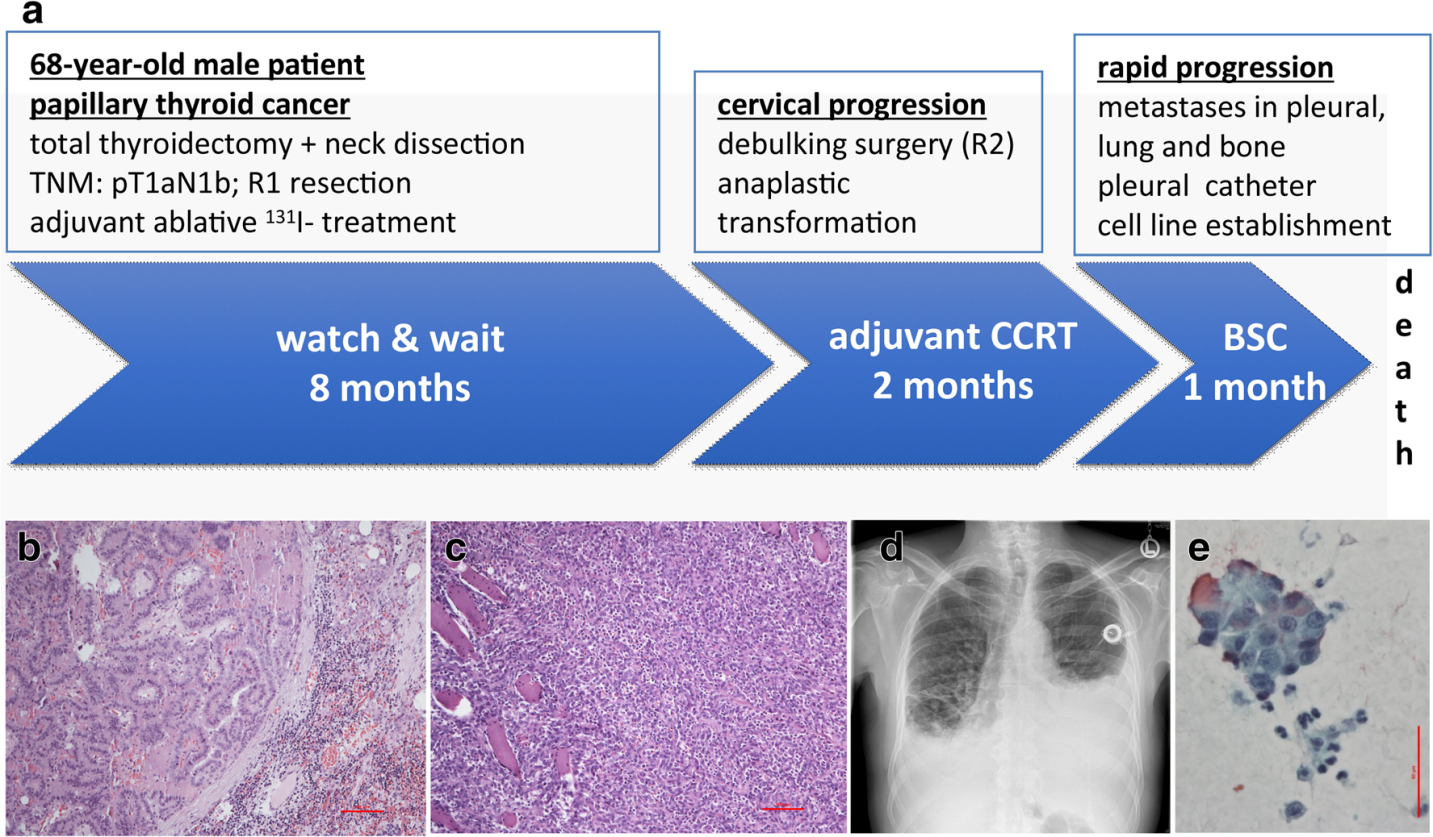
Patient clinical history. **a** Timeline of the patient’s treatment course. The patient received radical surgery followed by adjuvant ablative ^131^I radioiodine treatment and 8 months of watch and wait strategy. When the tumor locally relapsed the patient underwent debulking surgery and received adjuvant concurrent chemoradiotherapy (CCRT) for two months. The tumor rapidly progressed and best supportive care (BSC) was provided. **b** Paraffin embedded section stained with hematoxylin-eosin from a lymph node metastasis of the papillary thyroid tumor at the time of thyroidectomy. Bar represents 100 μm. **c** Paraffin embedded section stained with hematoxylin-eosin from the anaplastic tumor at the time of cervical progression. Bar represents 100 μm. **d** Chest radiograph at the time of tumor dissemination showing accumulating pleural effusions. **e** Positive Papanicolaou (PAP) staining on cytospin slide of the pleural effusion sample confirming malignancy. Bar represents 60 μm

A 68-year-old male patient was initially diagnosed with papillary thyroid cancer and subsequently underwent total thyroidectomy combined with neck dissection (Fig. 1a, b). Lymph node metastasis and R1 resection (microscopically positive tumor margin) was identified. Thus, the patient received adjuvant ablative high-dose ^131^I- treatment followed by a “watch and wait” strategy. Eight months later, the tumor locally relapsed and debulking surgery was performed. Histopathological analysis revealed anaplastic dedifferentiation (Fig. 1c), the cells lost TTF1 and thyroglobulin expression while retained CK18 and PAX8 positivity (Online Resource 2). Two months of concurrent adjuvant chemo-radiotherapy was administered. However, the tumor rapidly progressed with pleural, lung and bone metastases and a supportive pleural catheter to drain accumulating pleural effusions was applied. The pleural effusion (Fig. 1d) was proven malignant (Fig. 1e) and a cell line was established. The patient finally succumbed to the disease eleven months after initial diagnosis.

### Tumorigenicity of the Novel Cell Line (PF49)

**Fig. 2:**
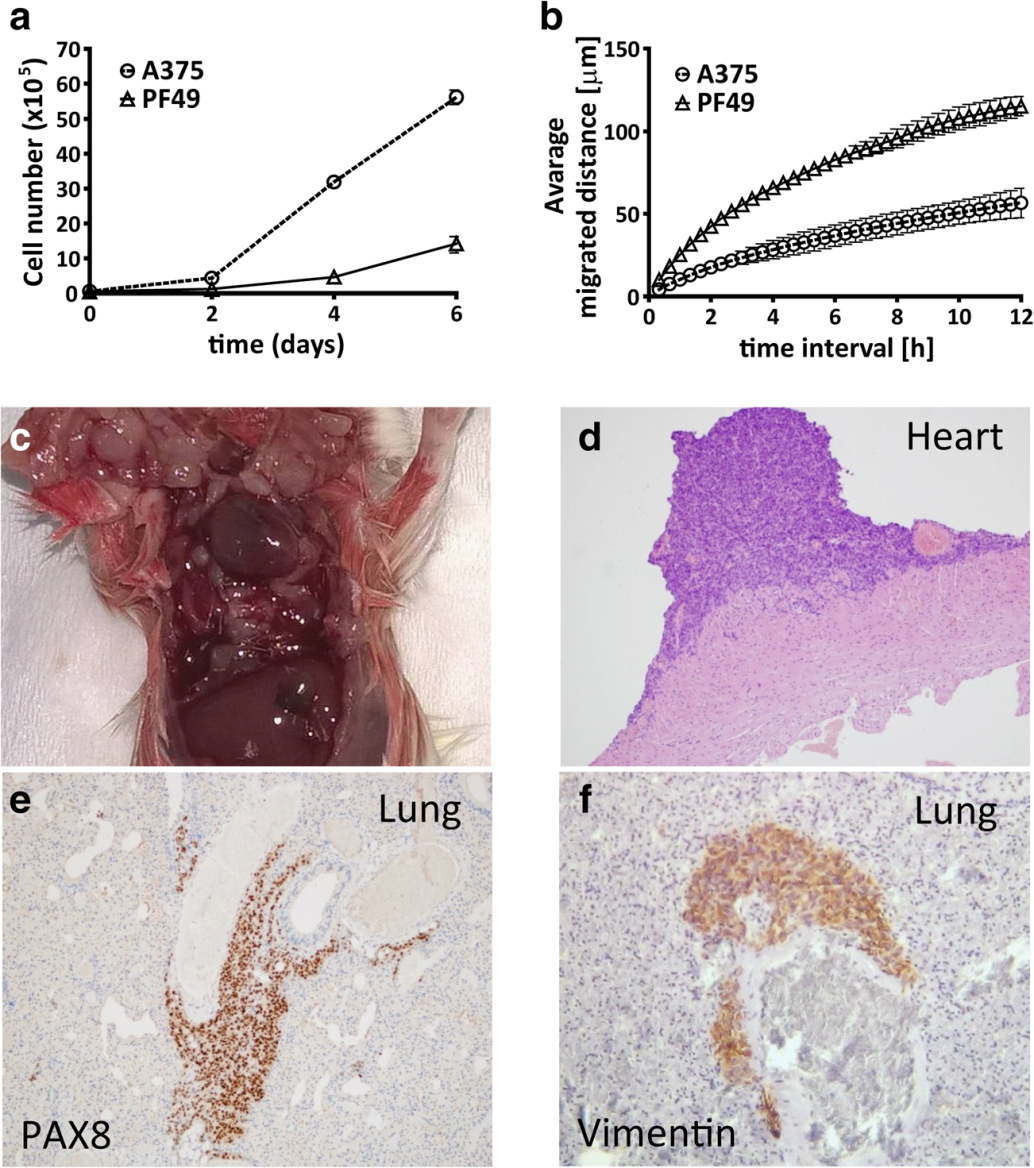
Proliferative, migratory and invasive capacity of PF49 cells. **a, b** Proliferation rate and migratory capacity of PF49 cells were compared with A375 melanoma cells. Cell number was determined after 2, 4 and 6 days. Migrated distance was measured with time-lapse video microscopy for 12 h. **c** In vivo orthotopic tumorigenicity and invasion was determined in a pleural carcinosis model. Paraffin embedded tumor sections were stained with hematoxylin-eozin **d**, PAX8 **e** and vimentin **f** antibodies. Bars represent means ±SE from two to three independent experiments

Since ATC cells often carry several genetic mutations, first we performed an oncogene panel analysis with next generation sequencing. We found that the cells have a BRAF V600E mutation and activating mutation in a TERT promoter (Online Resource 1). This is in good concordance with previous findings that these two mutations are common in this tumor type and show an association with advanced patient age [9]. Importantly, no RAS gene mutations, TP53 mutation or PI3K mutation were found in the cells, which are also often mutated in this cancer type [7]. Since ATC tumors usually grow rapidly and very invasively, we tested the proliferative and migratory capacity of the cells both in vitro and in vivo. We used the well characterized A375 melanoma cell line as reference that also carries a BRAFV600E mutation and highly motile [38]. We found that the proliferation rate of the PF49 cells was much slower than the A375 cells (Fig. 2a). However, time-lapse videomicroscopy showed that PF49 cells migrated much faster than the A375 cells (Fig. 2b, Online Resource 5). Furthermore, when we injected PF49 cells into the thoracic cavity of six 12-week-old SCID mice, tumors were formed on the surface of the pleura, in the lung parenchyma (Fig. 2c) and on the surface of the heart (Fig. 2d) within two weeks in all animals. The tumor cells also invaded the skeletal muscles of the chest wall. Immunohistochemical stainings demonstrated positivity for thyroid cell marker PAX8 and mesenchymal cell marker vimentin (Fig. 2e, f).

### Inhibition of the BRAF/MEK/ERK Pathway Reduces the Growth and Migration of PF49 Cells

**Fig. 3:**
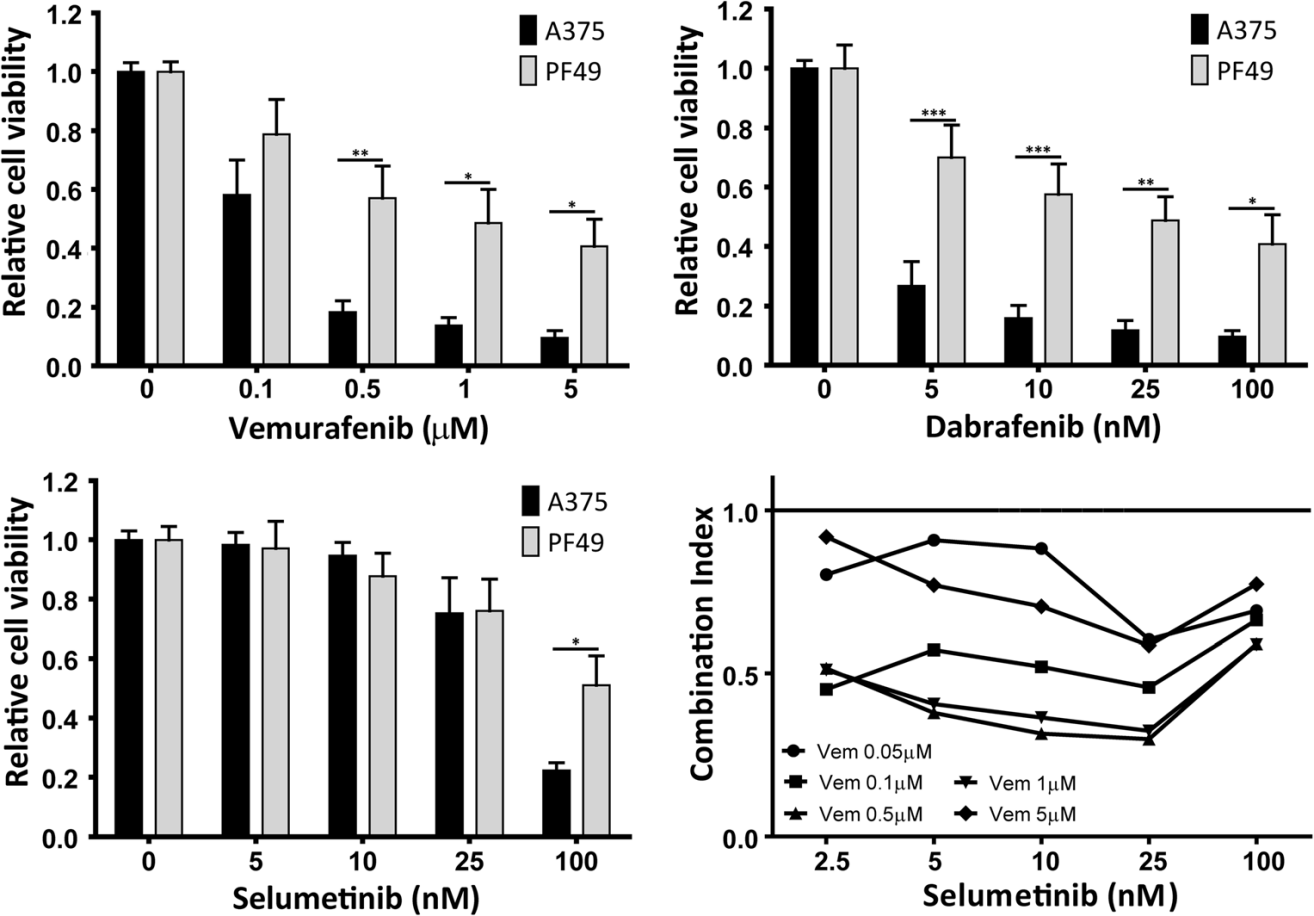
BRAF and MEK inhibitor sensitivity of PF49 cells in comparison to A375 melanoma cells. Cells were treated with increasing amount of BRAF inhibitor vemurafenib and dabrafenib and MEK inhibitor selumetinib alone and in combination. Cell viability was tested after 72 h with SRB assay. Statistical comparison was calculated by two-way ANOVA with Bonferroni posttests (*P < 0.05, **P < 0.01, ***P < 0.001). Bars represent means ±SE from two to three independent experiments. Combination index values less than 1 represent synergistic effect

**Fig. 4:**
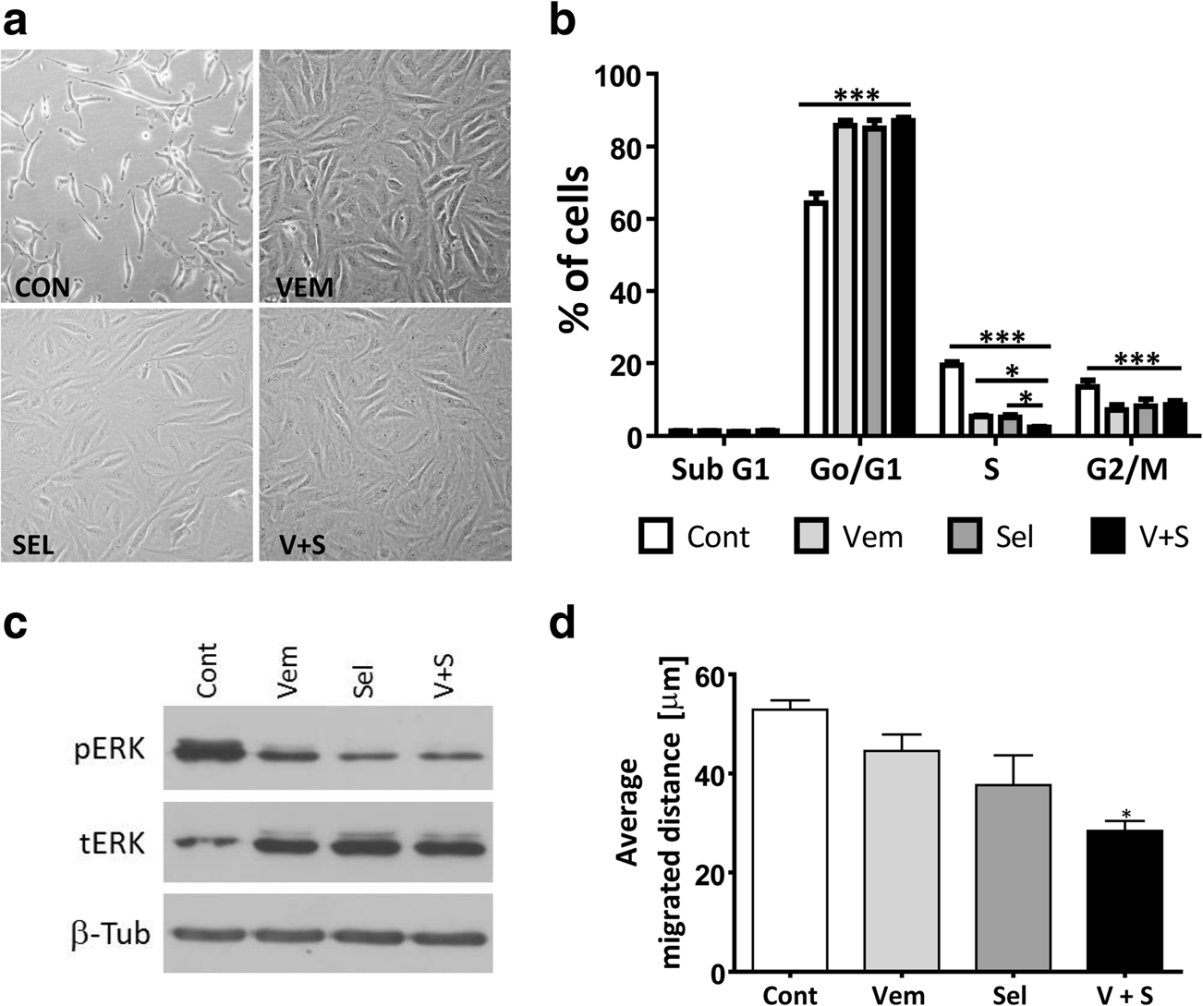
Mutant BRAF and MEK inhibitor treatment decreases the proliferation and migration of PF49 cells. **a, b** Cell morphology and cell cycle analysis was performed after 72 h-long treatment with 0.5 μM vemurafenib and 0.1 μM selumetinib alone and in combination. Phase contrast images (20x objective) show the morphological transition. One-way ANOVA followed by Tukey’s post hoc test was used to establish whether significant differences existed between groups. Differences were considered significant at *P < 0.05, **P < 0.01, ***P < 0.001. **c** Activation of ERK protein was analyzed by western blot after 72 h of treatment. **d** Average migrated distance was measured by time-lapse video microscopy in 3 h-long intervals between the 48 h and 72 h treatment period. Statistical comparison was calculated by one-way ANOVA with Dunn’s multiple comparison test (P < 0.05)

In metastatic melanoma the combination treatment of BRAF inhibitor dabrafenib and MEK inhibitor trametinib is used to treat patients with BRAF(V600E) mutant tumors successfully for many years [39]. Recently, the similar treatment was approved by the FDA for ATC patients [12]. We investigated the effect of these targeted therapeutic agents on PF49 cells. First, we performed Sulforhodamine B (SRB) toxicity assay with two mutant BRAF inhibitors vemurafenib and dabrafenib, and a MEK inhibitor selumetinib alone and in combination in PF49 and A375 melanoma cells (Fig. 3). A375 cells are widely used as a model sensitive to these treatments, thus we used these cells as a reference. We found that both the BRAF inhibitor and the MEK inhibitor treatment decreased the viability of PF49 cells but in a lesser extent as of the A375 cells. In PF49 cells combination treatment with various concentrations of the mutant BRAF inhibitor vemurafenib and MEK inhibitor selumetinib showed a strong synergistic effect on cell viability (Fig. 3). We analyzed the morphology of the cells after BRAF and MEK inhibition and found that the originally elongated spindle shaped cells demonstrated a more epithelioid morphology (Fig. 4a). Next, we performed cell cycle analysis after vemurafenib and selumetinib treatment alone and in combination. Our results show that both mutant BRAF and MEK inhibition strongly decreased the number of the cells in the synthesis and in the G2/M phases and this effect was significantly further increased in the S phase by the combination treatment (Fig. 4b). However, none of the treatments induced cell death. Immunoblot analysis showed that both BRAF and MEK inhibition decreased ERK activation in the cells as expected but combination of the two inhibitors did not further increase this effect (Fig. 4c). Since PF49 cells are very motile we analyzed the impact of BRAF and MEK inhibition on their migratory capacity. While both vemurafenib and selumetinib treatment modestly decreased the motility of the cells the combination treatment had a stronger effect (Fig. 4d). These results show that targeted therapy can effectively reduce the growth and the migration of the PF49 cell line; however, it fails to initiate cell death.

### HDAC Inhibitor Treatment Induces Cell Cycle Arrest Alone and in Combination of Conventional Chemotherapy

**Fig. 5:**
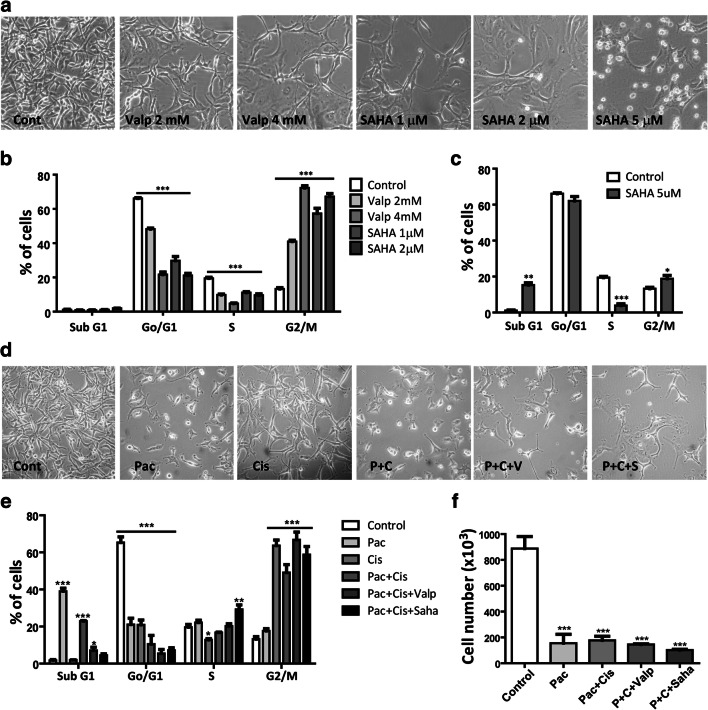
Both HDAC inhibitor treatment and chemotherapy induce cell cycle arrest in PF49 cells. **a** Cell morphology and **b, c** cell cycle analysis were performed after 72 h-long treatment with HDAC inhibitor valproic acid and SAHA, or with paclitaxel (10 nM), cisplatin (3 μM) alone and in combination with valproic acid (1 mM) or SAHA (1 μM) **d, e**. Pictures were taken with a phase contrast microscope (20x objective). One-way ANOVA followed with Dunn’s multiple comparison test was used to establish whether significant differences existed between groups. Differences were considered significant at *P < 0.05, **P < 0.01, ***P < 0.001. **f** Number of viable cells was determined after the treatments. One-way ANOVA followed by Tukey’s post hoc test was used to establish whether significant differences existed between groups

It was described earlier that HDAC inhibitor treatment can induce growth arrest and apoptosis in ATC cells. We used two broad spectrum HDAC inhibitors, namely suberoylanilide hyroxamic acid (SAHA) and valproic acid in different concentrations. We found that both treatments altered the morphology of the cells, but they did not obtain the epithelioid morphology as it was observed after BRAF or MEK inhibition (Fig. 5a). Cell cycle analysis showed that both valproic acid and SAHA treatment induced cell cycle arrest in the G2M phase in more than 50% of the cells, and in the case of SAHA this effect was already observed at a lower treatment concentration (1 μM) (Fig. 5b). SAHA initiated cell death in 15% of the cells but only at the highest treatment concentration (5 μM) (Fig. 5c). We also analyzed the effect of SAHA (2 μM) and valproic acid (2 mM) treatment on the migratory activity of the cells but we found no significant difference between the control and the treated cells (Online Resource 3).

We also tested the effect of conventional chemotherapy treatment on PF49 cells. We used paclitaxel alone and paclitaxel-cisplatin combination treatments as paclitaxel-carboplatin therapy was administered to the patient during the course of his disease. We also tested the effect of the combination of paclitaxel-cisplatin treatment with HDAC inhibition. We found that all the treatments strongly decreased the number of viable the cells (Fig. 5d, f). Cell death was induced in 40% of the cells by paclitaxel alone and in 20% of the cells by paclitaxel-cisplatin combination, however, the latter also induced cell cycle arrest in 50% of the cells. In the presence of HDAC inhibitors the ratio of dead cells decreased, however, more than 60% of the cells went into cell cycle arrest (Fig. 5e).

### HDAC Inhibitor Treatment Increases PD-L1 Expression of both PF49 and BHT-101 ATC Cell Lines

**Fig. 6:**
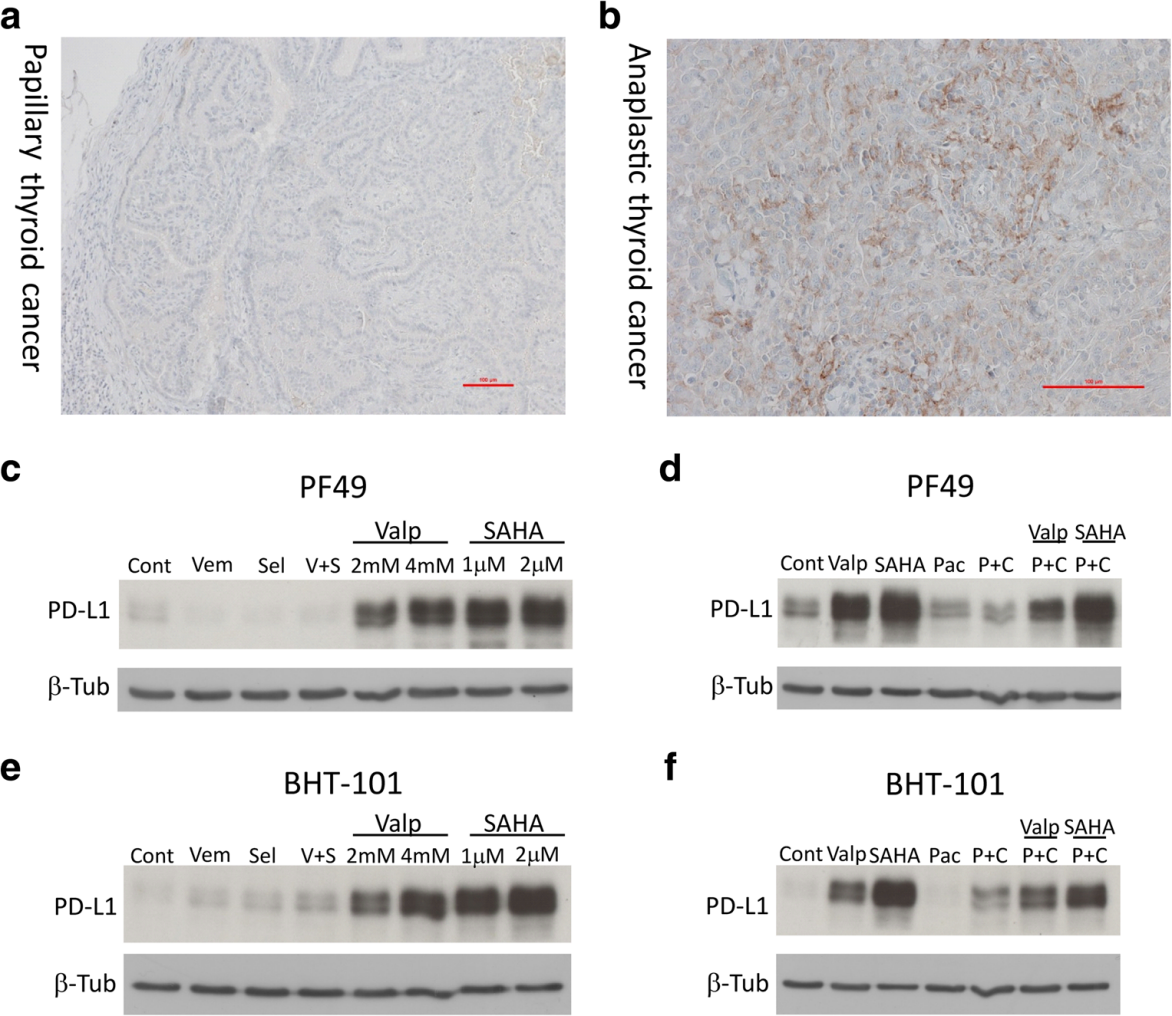
HDAC inhibitor treatment increases PD-L1 expression of both PF49 and BHT-101 ATC cell lines. **a** Paraffin embedded section stained with PD-L1 antibody from a lymph node metastasis of the papillary thyroid tumor at the time of thyroidectomy. Bar represents 100 μm. **b** Paraffin embedded section stained with PD-L1 antibody from the anaplastic tumor at the time of cervical progression. Bar represents 100 μm. **c-f** Expression level of PD-L1 protein was analyzed in PF49 and BHT-101 cell lines by western blot. Cells were treated with vemurafenib (0.5 μM), selumetinib (0.1 μM), paclitaxel (10 nM), cisplatin (3 μM), SAHA or valproic acid alone or in combinations for 72 h

Since PD-L1 mAb treatment is a potential novel modality in ATC [15], we analyzed the PD-L1 expression of the initially diagnosed papillary thyroid carcinoma and the later developed anaplastic thyroid carcinoma immunohistochemically. We found that the PTC was essentially negative for PD-L1 (CPS 0.75) (Fig. 6a), while the ATC was moderately positive (CPS 12) (Fig. 6b). We also investigated if targeted therapy, HDAC inhibition or chemotherapy influenced PD-L1 expression of the PF49 cells. We found that treatment with both SAHA and valproic acid robustly increased the PD-L1 expression of the PF49 cells at all treatment concentrations while mutant BRAF or MEK inhibition slightly even decreased it (Fig. 6c, Online Resource 4). Importantly, this effect of the HDAC inhibitors was also present when they were combined with paclitaxel-cisplatin while paclitaxel or paclitaxel-cisplatin alone caused no change in the PD-L1 expression of the cells (Fig. 6d). In order to investigate if PD-L1 expression can be induced by HDAC inhibitor treatment in other ATC cell lines as well, we performed the same treatment regimen also on BHT-101 cells. This cell line also carries the BRAF V600E and a TERT promoter mutation similarly to PF49. We found that both SAHA and valproic acid treatment strongly induced PD-L1 expression in BHT-101 cells (Fig. 6e, Online Resource 4). In this cell line, paclitaxel-cisplatin treatment alone also elevated PD-L1 expression moderately, which was further increased by combination with HDAC inhibitors (Fig. 6f). In contrast to PF49 cells, mutant BRAF or MEK inhibition slightly increased the PD-L1 expression in BHT-101 cells. Of note, HDAC inhibitor treatment also increased PD-L1 expression in A375 melanoma cells (Online Resource 6).

## Discussion

While patients with differentiated thyroid cancers have a good prognosis with long term survival when receiving the standard therapy, anaplastic thyroid cancer has a really poor outcome due to its high metastatic capacity and poor response to both radioiodine and chemotherapeutic treatments. For this reason, new translational models and treatment modalities are still needed in this tumor entity. In the present study, we established a new anaplastic thyroid cancer cell line (PF49) from the pleural effusion of a 68-year old male patient. ATCs metastasize to the pleura in around 20% of the patients and in some cases can also provoke malignant pleural effusion [32, 40]. The patient was first diagnosed with papillary thyroid cancer that transformed to anaplastic thyroid cancer during an 8-month period. We identified a BRAF(V600E) mutation and a TERT promoter mutation in the PF49 tumor cells and found that these mutations were already present in the primary PTC. BRAF(V600) is present in approximately 25% of the ATC cases and it is found in a similar ratio in the already established ATC cell lines [6, 41]. TERT promoter mutation was described in 40% of the ATC cases and papillary carcinomas that carry a TERT promoter mutation are more likely to transform to ATC [7, 42]. The cells showed positivity for thyroid cell marker transcription factor molecule PAX-8 and cytokeratin 18, however, they were negative to differentiated thyroid tumor markers such as thyroglobulin and thyroid transcription factor-1 (TTF-1) which is in good accordance with previous observations in other ATC cell lines [41, 43, 44]. Interestingly, ATC cell lines show a strong correlation with ATC tissue samples in their mRNA expression profile while differentiated thyroid cancer cell lines show a stronger difference in their DNA replication regulation compared to tumor tissue [45]. We found that the ATC cells have a very strong migratory capacity in vitro and demonstrated that in a pleural carcinosis model their invasive capacity is also very high. Accordingly, this orthotopic mouse model could be a valuable tool to test new treatment modalities against advanced ATC [46].

Since ATCs have very diverse genetic background, treatment regiments specific for a given set of mutations are likely to be developed in the future. BRAF kinase mutation is found in 60% of PTC and 20–40% in ATC. Since BRAF mutant PTCs are often precursors for ATC, a transgenic mouse model was developed with Cre-regulated BRAFV600E mouse and a conditional Trp53 allelic series [47]. In these animals, combined treatment with mutant BRAF inhibitor PLX4720 and MEK kinase inhibitor PD0325901 strongly increased the survival of the animals. Furthermore, in orthotopic thyroid mouse models, PLX4720 could sufficiently decrease tumor growth and lung metastases in both early and late-intervention models [48, 49]. Additionally, in a phase II, open label trial on 16 patients with BRAF(V600E) mutant ATC BRAF inhibitor dabrafenib and MEK inhibitor trametinib was used that generated high response rate (69%) and prolonged survival during the 47 week-long follow up [12]. In good accordance with this results, we found that the PF49 cells which are also carrying a BRAF(V600) mutation, responded with decreased growth and reduced migration to both BRAF and MEK inhibitor treatments and combination of these treatments could further increase this effect. However, the inhibitory effect was weaker than in the melanoma cell line A375 and it did not induce cell death. The PF49 cells also carry a TERT promoter mutation that in turn is associated with anaplastic transformation of PTC [42]. It also shows a correlation with extrathyroidal extension, stage IV tumors and advanced age [50]. Combined presence of TERT and BRAF mutations was shown to even further increase the risk of disease related death in PTCs [51]. TERT protein expression was associated with the expression of EMT markers in PTC tissue samples and inhibition of TERT reversed EMT and decreased the migration and metastasis of PTC cells [50]. Nevertheless, it is still unknown, if the effectiveness of the combined BRAF/MEK inhibitor treatment is influenced by the TERT promoter status of the tumors.

HDAC inhibitors form a chemically diverse group of compounds and their anti-tumor effect has been long described such as inhibition of tumor cell proliferation and induction of apoptosis. We tested the effect of two structurally different HDAC inhibitors on PF49 cells SAHA and VPA and found they both induce cell cycle arrest. While SAHA caused cell cycle arrest already at a lower concentration it could initiate apoptosis only at the high 5 μM concentration and VPA did not induced apoptosis in these cells at all. HDAC inhibition did not affect the migratory capacity of the cells although an anti-migratory effect of HDAC inhibition was described in other cancer cells before [52, 53]. Importantly, HDAC inhibition induced robust PD-L1 expression in PF49 cells. Combination treatment of HDAC inhibitors with immune checkpoint inhibitors is widely investigated recently [54] and gives promising results in triple negative breast cancer [55] and in melanoma cells [56, 57]. In ATC, PD-L1 and PD1 expression of tumor cells and tumor infiltrating immune cells were previously investigated. ATC tumor cells showed a varying PD-L1 expression with a subset of tumors with high expression and the presence of tumor infiltrating PD-L1 positive lymphocytes was also frequent [13]. One study also examined but found no significant association between BRAF(V600E) mutation of the cells and PD-L1 expression on the tumor cells [58]. We found that the original papillary thyroid carcinoma was negative for PD-L1 staining, while the anaplastic carcinoma was moderately positive. This is in good accordance with the results of Cantara et al. [15], who found that normal thyroid and differentiated TC samples were negative, while 70–90% of ATC samples were positive for PD-L1. Gene amplification might drive increased expression of proteins that control immune evasion. PD-L1 copy number alternation was analyzed in a large panel of solid tumors and PD-L1 was amplified in 9 out of 177 ATC cases [59]. This is a relatively high frequency compared to other malignancies and might contribute to the effectiveness of check point inhibition in this tumor type [60]. These results reinforce that immunotherapy could be effective against ATC. We found that BRAF inhibitor and MEK inhibitor treatment slightly decreased PD-L1 expression in PF49 cells but somewhat increased it in in BHT-101 cells. It was found that when transgenic BRAFV600E/WT P53*−/−* mice were treated with BRAF inhibitor PLX4720 and PD-L1 antibody the combination treatment strongly decreased tumor volume compared to single treatment through increased anti-tumor immune response [61]. Currently, there is an ongoing phase II clinical trial where BRAF inhibitor vemurafenib, MEK inhibitor cobimenitib and anti-PD-L1 inhibitor atezolizumab are used in combination to treat patients with BRAF V600E mutated ATC. Early results show that this treatment can reduce tumor volume in an extent that afterwards R0 or R1 surgery is possible [62] .

Chemotherapy plays still a major role in ATC treatment regimens. Paclitaxel alone, paclitaxel-carboplatin combination and doxorubicin are administered most commonly [63]. Among these paclitaxel was found to be the most effective in vitro and its combination with vinorelbine and gemcitabine further increased the antitumor effect [64]. We tested the paclitaxel and cisplatin treatment separate and in combination in PF49 cells while the patient was treated with paclitaxel-carboplatin previously. We found that paclitaxel single treatment initiated cell death most strongly, and combination with cisplatin initiated more cell cycle arrest but the number of viable cells was similar after the two treatments. Further combination of paclitaxel and cisplatin with low concentration valproic acid (1 mM) or SAHA (1 μM) reduced the amount the viable cells similarly but strongly increased PD-L1 expression of the tumor cells. It was shown earlier that VPA enhanced the anti-apoptotic effect of paclitaxel [65]. However, in a phase II clinical trial combined use of VPA and paclitaxel was compared to paclitaxel alone and no improvement in the clinical outcome was found [29]. Our results indicate that VPA or SAHA may enhance the effectiveness of PD-L1 antibody treatment in ATC patients either alone or in combination with chemotherapeutic treatment. Further investigations are required on larger patient cohorts to determine PD-L1 expression level both in primary and metastatic tumor samples and in ATCs with different mutational background.

## Electronic supplementary material

ESM 1 (MPEG 6828 kb)

ESM 2 (PDF 496 kb)

## Data Availability

Upon reasonable request the data and the cell line is available from the corresponding author.
