# Supplementary material for: HDAC Inhibition Induces PD-L1 Expression in a Novel Anaplastic Thyroid Cancer Cell Line
Source: Pathol Oncol Res. 2020 Jun 26;26(4):2523–35. doi: 10.1007/s12253-020-00834-y (PMC7471186; doi:10.1007/s12253-020-00834-y)
Supplement: Supplementary file 2 — (PDF 496 kb) [file 12253_2020_834_MOESM2_ESM.pdf]

Supplementary Information (Pathology & Oncology Research)

HDAC inhibition induces PD-L1 expression in a novel anaplastic thyroid cancer cell line

Luca Hegedűs, Dominika Rittler, Tamás Garay, Paul Stockhammer, Ildikó Kovács, Balázs Döme, Sarah Theurer, Thomas Hager, Thomas Herold, Stavros Kalbourtzis, Agnes Bankfalvi, Kurt W. Schmid, Dagmar Führer, Clemens Aigner, Balázs Hegedűs

Corresponding author: Balázs Hegedűs, Department of Thoracic Surgery, Ruhrlandklinik, University Duisburg-Essen, Essen, Germany, Tel.: 49 201 4334665, Fax: 49 201 4334019, E-mail: balazs.hegedues@rlk.uk-essen.de

Online Resource 1. The mutational analysis of the PF49 cell line was performed by NGS.

**PF49 cell line**	**Mutational status**
STK11	WT
RET	WT
PIK3 CA	WT
PDGFR A	WT
BRAF	V600E
EGFR	WT
ERB B2	WT
FGFR1,3	WT
HRAS	WT
ID1	WT
IDH2	WT
KIT	WT
KRAS	WT
MET	WT
NRAS	WT
TP53	WT
TERT promoter status	Mutation chr5: 1295228C>T (hg19)

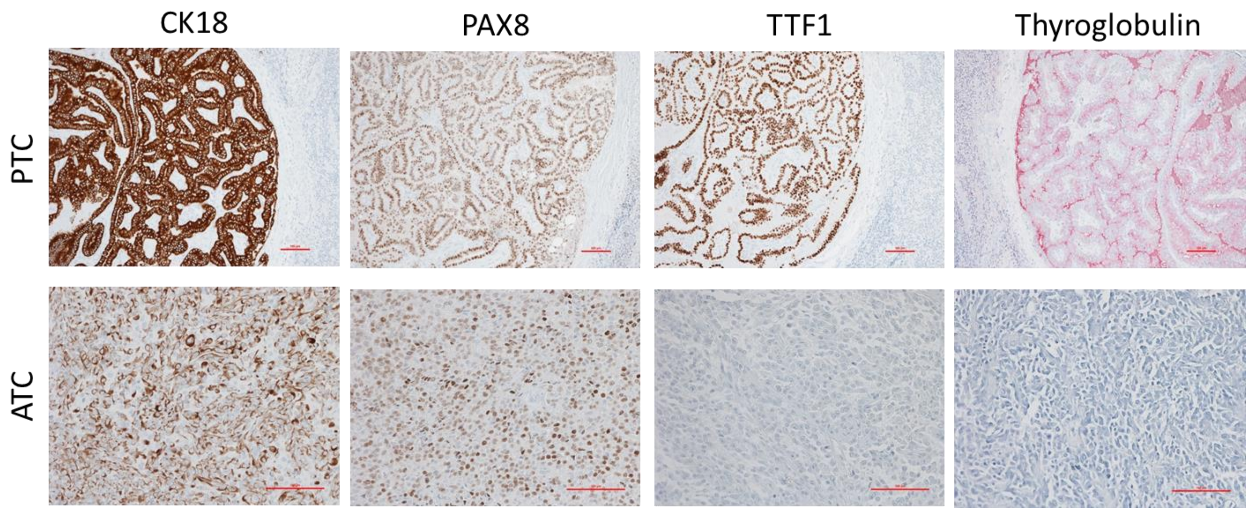

Online Resource 2. Paraffin embedded sections from the lymph node metastasis of the papillary thyroid tumor and from the anaplastic tumor were stained with PAX8, CK18, TTF1 and thyroglobulin antibodies. Bars represent 100 μm.

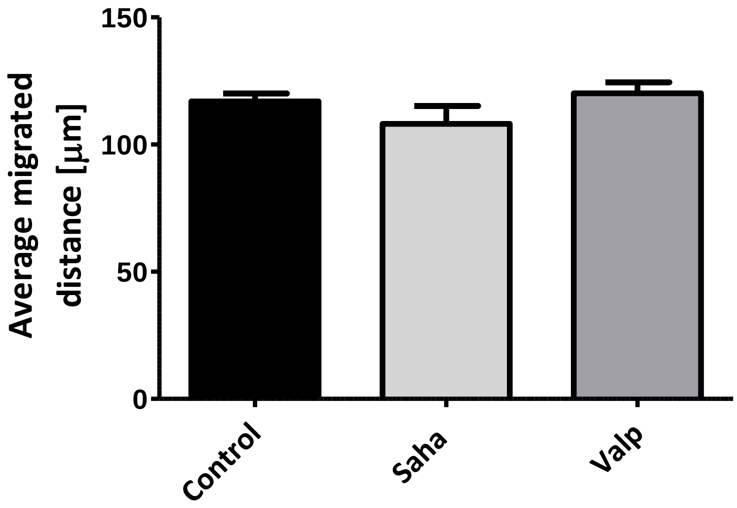

Online Resource 3. PF49 cells were treated with SAHA (2 μM) or valproic acid (2 mM) for 72 hours. Average migrated distance in 6 hour-long intervals was measured by time-lapse video microscopy between 48 and 72 hours after treatment.

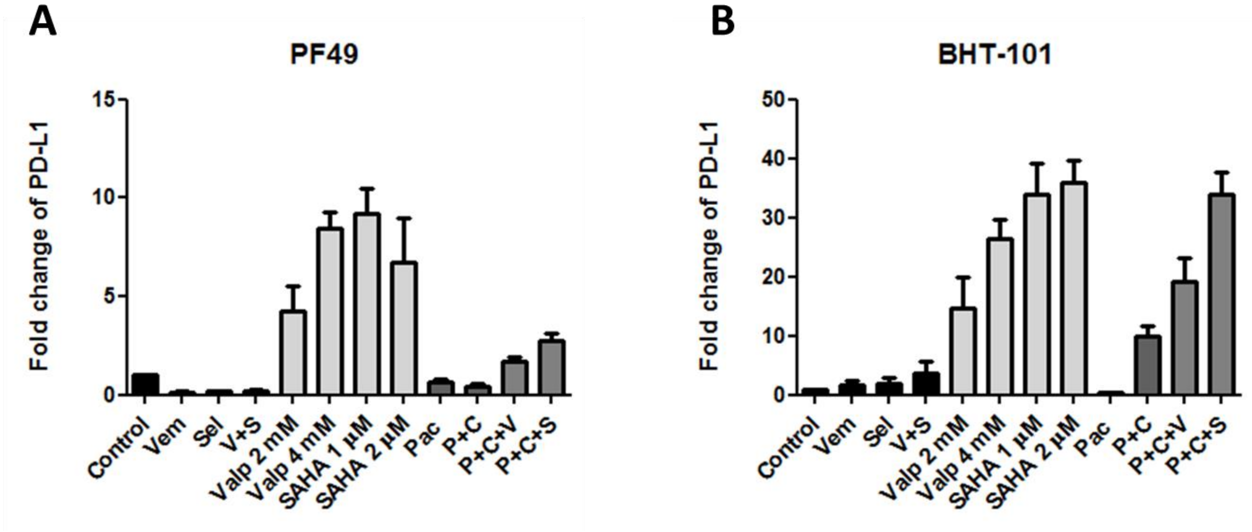

Online Resource 4. PF49 (A) and BHT-101 (B) cells were treated with vemurafenib (0.5 μM), selumetinib (0.1 μM), paclitaxel (10 nM), cisplatin (3 μM), SAHA or valproic acid alone or in combinations for 72 hours. Expression level of PD-L1 protein was analyzed by western blot. Bars represent means ±SE from two to three independent experiments.

Online Resource 5. Time-lapse videomicroscopy of untreated PF49 cells.

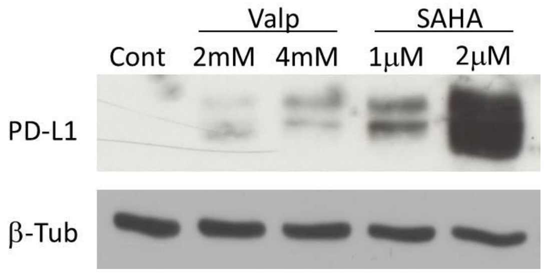

Online Resource 6. A375 cells were treated with SAHA or valproic acid for 72 hours. Expression of PD-L1 protein was analyzed by western blot.
